# Self-reported and informant-reported memory functioning and awareness in patients with mild cognitive impairment and Alzheimer´s disease

**DOI:** 10.1007/s40211-016-0185-y

**Published:** 2016-06-13

**Authors:** Martina Rios Silva, Doris Moser, Melanie Pflüger, Gisela Pusswald, Elisabeth Stögmann, Peter Dal-Bianco, Eduard Auff, Johann Lehrner

**Affiliations:** Department of Neurology, Medical University of Vienna, Währinger Gürtel 18–20, 1097 Vienna, Austria; Faculty of Psychology, University of Vienna, Vienna, Austria

**Keywords:** Anosognosia, Awareness, Subjective memory assessment, Alzheimer’s disease, Mild cognitive impairment, Anosognosia, Awareness, Subjektive Gedächtnisbeschwerden, Leichte kognitive Störung, Alzheimer Demenz

## Abstract

**Background:**

Awareness of subjective memory is an important factor for adequate treatment of patients with mild cognitive impairment (MCI) and Alzheimer’s disease (AD). This study served to find out whether awareness of subjective memory complies with objective performance, if differences in awareness are observed longitudinally and whether decrease of awareness can serve as a predictor of AD in MCI patients.

**Methods:**

Thirty-four patients with MCI seeking help in a memory outpatient clinic were included. All participants underwent thorough neuropsychological examination. Awareness of subjective memory was obtained by calculating difference scores between patient and informant ratings on a 16-item questionnaire concerning complaints about loss of memory in every-day life. Retesting was performed after a mean follow-up period of 24 months.

**Results:**

Whole group analyses showed that awareness remained relatively stable across time. Self-reported memory complaints correlated with episodic memory at baseline and with performance on a language task at follow-up. Retests displayed decrease of awareness. At group level differences in awareness between both times of assessment were not significant for MCI and MCI patients converting to mild AD at follow-up. The predictive value of awareness was low.

**Conclusions:**

Awareness of subjective memory deficit is linked to episodic memory function and decreases with decline of cognitive ability. Further studies evaluating predictive power of awareness of subjective memory should include a larger patient sample.

## Zusammenfassung

**Grundlagen:**

Die Einsicht (Awareness) hinsichtlich der Erinnerungsleistung ist ein wichtiger Faktor für eine angemessene Behandlung von Patienten mit leichter kognitiver Beeinträchtigung (MCI) und Alzheimer-Krankheit (AD). Ziel der vorliegenden Studie war herauszufinden (i) ob subjektive Gedächtnisleistung und objektive Gedächtnisleistung assoziiert sind, (ii) ob sich die Einsicht (Awareness) hinsichtlich der Erinnerungsleistung mit der Zeit verändert und (iii) ob die Einsicht (Awareness) hinsichtlich der Erinnerungsleistung ein Prädiktor für die Entwicklung einer Demenz ist.

**Methodik:**

Vierunddreißig Patienten mit MCI, die Hilfe in einer universitären Gedächtnisambulanz suchten, wurden in die Studie eingeschlossen. Alle Teilnehmer wurden einer ausführlichen neuropsychologischen Untersuchung unterzogen. Die Einsicht (Awareness) hinsichtlich der Erinnerungsleistung wurde durch Berechnung der Differenzwerte zwischen Patient und Informant Bewertung auf einem 16-Punkt-Fragebogen hinsichtlich Gedächtnisbeschwerden im täglichen Leben erhalten. Eine Kontrolluntersuchung wurde nach einer mittleren Nachbeobachtungszeit von 24 Monaten durchgeführt.

**Ergebnisse:**

Die Analyse zeigte, dass die Einsicht (Awareness) hinsichtlich der Erinnerungsleistung relativ stabil über die Zeit blieb. Selbst berichtete Gedächtnisbeschwerden korrelierten mit der episodischen Gedächtnisleistung zu Beginn der Studie und mit der Leistung bei einer Sprachaufgabe bei der Nachuntersuchung. Wiederholungsprüfungen angezeigt Rückgang des Bewusstseins. Der prädiktive Wert der Einsicht (Awareness) hinsichtlich der Erinnerungsleistung in Bezug auf die Demenzentwicklung war gering.

**Schlussfolgerungen:**

Einsicht (Awareness) hinsichtlich der Erinnerungsleistung ist mit episodischer Gedächtnis-Funktion verknüpft und nimmt mit Abnahme der kognitiven Fähigkeiten ab. Weitere Studien zur Vorhersagekraft der Einsicht (Awareness) hinsichtlich der Erinnerungsleistung sollten eine größere Patientenstichprobe umfassen.

## Introduction

 Elderly people often experience cognitive decline with aging. The reasons for cognitive dysfunctions can range from physiological mild forgetfulness described by many older individuals to mild cognitive impairment until the severe effects of Alzheimer’s disease [1]. Many patients with considerable subjective memory complaints (SMC) seek help at a memory outpatient clinic, and complaints increase from cognitive healthy elderly to patients with mild cognitive impairment (MCI) [2]. On the other hand, many patients with mild cognitive impairment (MCI) and Alzheimer’s disease (AD) do not recognize cognitive, functional or behavioral impairment [3]. But this anosognosia [4] can have serious effects on health, because patients eventually deny adequate treatment due to their unawareness of deficits. Daily functioning may be compromised, because they lack adequate judgement of situations [5].

Subjective memory complaints (SMC) are supposed to be an early symptom of dementia and therefore are often applied in the diagnostic process [6]. Cognitive decline is often accompanied by a change of awareness of deficits. Even in the earliest stages of AD and actually MCI, insight can be impaired and this lack of anosognosia is most common in severe AD [4, 7]. Vogel et al. [7] compared awareness of cognitive deficits in patients with MCI and mild AD and came to the conclusion, that impaired awareness was equally frequent in both groups with individual significant heterogeneity in the degree of impaired insight. Different studies regarding awareness in MCI demonstrate a great variability among this patient group. While some people with MCI show limited awareness, others seem to overestimate their dysfunction (also declared as reflecting heightened or hyper-awareness). Depressive symptoms may have negative influence on the expression of awareness and may increase negative attributions, making memory problems seem more severe than they are [2, 8]. Sevush and Leve [9] found, that denial of deficits might protect against depression in Alzheimer’s disease, because unawareness was inversely related to depressed mood. Therefore informants are often involved in diagnosis and assessment of subjective memory awareness [10].

A review concerning self and informant reports in MCI patient illustrates that informant ratings display greater loss of cognitive competency and everyday functional ability and a greater correlation with objective measures of patient cognitive performance and characteristics of probable conversion to dementia [10]. Ecklund-Johnson and Torres [4] reviewed studies regarding unawareness of deficits in AD and found that unawareness of deficits progresses over time and awareness discrepancies between patients and their caregivers increase. Although informant reports might be influenced negatively by caregiver burden, informant ratings have turned out to be a strong predictor of an underlying dementia. The authors also conclude that memory deficits alone neither explain nor predict unawareness of deficits in AD. Brain correlates of unawareness in dementia were mainly detected in frontal and tempo-parietal regions, but further research is needed [3].

The current research succeeds a recent cross-sectional study by Lehrner et al. [11]. They concluded that awareness decreases along the nonamnestic MCI → amnestic MCI → AD continuum. The main objective of the present longitudinal study was to find out, whether awareness of subjective memory in patients with MCI has predictive value for future conversion to AD. The methods were based on precedent studies using additional informant ratings [7]. The first aim was to explore correlations between subjective memory assessment and objective results of neuropsychological testing. Correlation analyses comprised neuropsychological test results and demographic data. Two variables measuring depressive symptoms were also included in order to examine the influence of depression on awareness [2, 8]. The second aim was to figure out differences in awareness longitudinally. We hypothesized that small differences across time would indicate an intact awareness system, while large differences would reveal loss of awareness. It was assumed that decline of cognitive performance combined with decline of awareness between both times of assessment would imply anosognosia as observed in inherent dementia [4]. The final intent was to find out, if unawareness of memory deficits could serve as a predictor of future AD in patients with MCI.

## Methods

### Subjects and procedure

The current study is part of a larger research project, the Vienna Conversion to Dementia Study. The data of this quasi-experimental longitudinal study were collected at the Department of Neurology of the Medical University Vienna. The study protocol has been approved by the Ethical Committee of the Medical University of Vienna and written informed patient consent to perform this study was received.

### Patients

The study is based on a sample of 34 consecutive patients aged 50 years or older, who came to the memory outpatient clinic of the Medical University of Vienna due to self or informant reported memory problems and who fulfilled the inclusion criteria. They were either referred by physicians, by the Department of Neurology, or they were self-referrals. All patients went through clinical examination and neuropsychological testing. Exclusion criteria were evidence of stroke, traumatic head injury in the past, or other neurological disease, current psychiatric diagnosis according to ICD-10 [12] and any medical condition leading to cognitive deterioration. However, patients with depressive symptoms suggesting “depressive episode” according to ICD-10 were included, because these frequently appear in elderly people [2].

By means of a multi-group design, patients were divided into the subgroups aMCI, naMCI and AD after completion of the evaluation. MCI-subtypes were chosen according to Petersen et al. [13], using a cut-off score of 1, 5 standard deviations below age and education. AD was diagnosed according to DSM-IV criteria [14] and the NINCDS-ADRDA [15]. Additional informant ratings about subjective assessment of memory were included in the study.

## Measures

### Neuropsychological Test Battery Vienna (NTBV)

After a detailed anamnesis interview including standard questions about memory functions and a short survey of the accompanying person, the Mini Mental Status Examination (MMSE) [16] was performed. Additionally, all patients were subjected to the Neuropsychological Test Battery Vienna (NBTV) [17]. Patients with a MMSE score of less or equal 23 were excluded from the study. The NBTV includes tests for attention, executive functioning, language, and memory domains with corresponding z‑scores and was found to have very good discrimination power in detecting dementia [17]. Attention is assessed using the Alters-Konzentrationstest (AKT) [18], a geriatric cancellation test, the digit symbol subtest of the German WAIS-R [19] and the symbol counting subtest from the cerebral insuffiency test (C.I.) [20]. The Trail Making Test B [21] and the score difference of the Trail Making Test A and B are also used to measure attention. The Trail Making Test A [21] is applied to investigate the executive function, which was also assessed by the Five-Point-Test [22], the Maze Test and the Stroop Test from the NAI Battery [23] and the interference test from the C.I. [20]. Lexical verbal fluency is investigated by naming as many words beginning with the letters b, f, and l that come to mind within one minute for each task. Language functions are tested by use of a verbal fluency tasks and a confrontation naming task [24]. Semantic and verbal fluency is assessed by naming as many animals, supermarket items and tools that came to mind within one minute for each task. The modified Boston Naming Test (mBNT) [25] is submitted for testing naming capabilities. Episodic memory is applied using the Verbal Selective Reminding Test (VSRT) [26] with the subtests of immediate recall, total recall, delayed recall, and recognition.

### Cognitive functioning (intelligence-quotient; IQ)

The Wortschatztest (WST) [27], a standardized vocabulary test was applied to assess crystallized intelligence of all participants. The WST is a standardized vocabulary test, which is commonly used to examine verbal comprehension in patients with brain damage or dementia. See Table 1 for results.

**Table 1:** Demographic characteristics of basic sample (*n* = 34)

	naMCI	aMCI
*n*	15	19
Age	70 (55–79)	71 (57–85)
Male/Female	8/7	9/10
Education	8 (5–15)	8 (8–17)
MMSE	28 (26–30)	27 (24–30)
BDI	10 (2–19)	5 (0–27)
GDS	3 (0–8)	2 (0–10)
FAI Self	2.56 (1.38–3.38) ^a^	2.88 (1.38–4.06) ^b^
FAI Informant	3.12 (1.31–4.62)	2.84 (1.63–4.38)
Awareness	−0.61 (−2.31–1.75) ^a^	−0.20 (−2.50–2.25) ^b^

Median (Range); *MMSE* Mini Mental State Examination, *BDI* Beck Depression Inventory, *GDS* Geriatric depression scale, *FAI* Forgetfulness Assessment Inventory

^a^ *n* = 14

^b^ *n* = 18

### Assessment of subjective memory complaint

Subjective memory problems were assessed by use of the Forgetfulness Assessment Inventory (FAI) scale [2]. The questionnaire consists of 16 questions concerning subjective memory difficulties in daily life within the past four weeks. Measurement is based on a 5 point Likert scale. Questions are e. g. “How often did you have problems during the past 4 weeks remembering … e. g. names of people. 1 = never, 2 = rarely, 3 = sometimes, 4 = often, 5 = very often.” Other items include memory difficulties concerning telephone numbers, faces, birthdays or shopping lists (for specific items see Appendix, Table A). The average of all 16 items represents a global score of memory complaints, which ranges from 0 to 5. Higher scores indicate worse subjective memory performance and greater complaints. Accompanying persons were asked to fill in the informant version of the FAI in order to appraise memory functioning of the concerned patient (www.meduniwien.ac.at/kpfg). The FAI has been shown to have good psychometric test criteria [2]. See Table 1 for results.

### Assessment of depressive symptoms

Depressive symptoms were tackled by use of the Beck Depression Inventory (BDI-II) [28] and the Geriatric Depression Scale (GDS) [29]. The BDI-II consists of 21 items that ask about how often one felt certain ways within the past two weeks, rating on a four-point scale. Scores above 13 are consistent with clinical depressive symptoms. The BDI-II has been successfully used for screening depressive symptoms [36]. The GDS is a 15-item self-assessment questionnaire, which was developed to identify depression in the elderly. Participants are asked how they felt over the past week. The questions can be answered yes or no, with higher scores representing higher depressive symptoms. Scores above 5 are consistent with clinical depressive symptoms. The GDS has been successfully used for screening depressive symptoms [37]. See Table 1 for results.

### Awareness

Awareness scores were assessed by subtraction of FAI informant rating scores from self rating scores. Positive signs imply that participants underestimated their memory functions in relation to their caregivers, negative signs suggest patients’ overestimation. It is assumed that higher discrepancy scores indicate greater unawareness [7, 8]. See Table 1 for results.

### Statistical Methods

Demographic variables are described by median and range due to skewed distribution. At first step, correlations were calculated to compare whole-group FAI self, informant and awareness scores with objective performance measures and non-cognitive factors. Spearman correlations were chosen, because most of the NTBV-variables were not normally distributed. Second, whole-group comparisons of subjective memory assessment between baseline and follow-up were made using Pearson correlations. Normal distribution was verified by Kolmogorov-Smirnov test. At third step, differences across time were calculated at group-level. Wilkoxon signed-rank test was used to calculate differences across time within the MCI and AD diagnoses groups, because the assumptions of equal group-sizes and normal distribution for calculating repeated measures ANOVA were violated. Finally, Receiver operator characteristics analysis (ROC) was conducted in order to evaluate predictive value of FAI variables. Statistical analyses were performed using SPSS (version 22). The reported *p*-values result from two-tailed tests and are statistically significant at the level of *p* < 0.05. Effect-sizes are reported according to Cohen [30], interpreting a correlation coefficient of r = 0.10 as small effect, r = 0.30 as medium effect and r = 0.50 as large effect.

## Results

### Descriptive data

A total of 34 patients between 55 and 85 years (*Mdn* = 70 years) were assessed at a 2-year follow-up (*Mdn* = 28 months). Altogether, 17 males (50 %) and 17 females (50 %) were included in the study. Mean duration of formal education was 8 years, ranging from 5 to 17 years. At the baseline 34 patients were diagnosed as MCI, 19 of them were classified as aMCI, 15 as naMCI according to Petersen [13]. See Table 1 for details. At follow up 13 patients (38,2 %) converted to AD, 4 of them were male, 9 female. Table 2 presents conversion rates including the MCI subgroups aMCI and naMCI. Within the group of baseline diagnosis aMCI, 11 people (57,9 %) turned to AD, while only 2 of the initial naMCI patient group (13,3 %) were diagnosed as AD at follow up.

**Table 2:** Rates of conversion in aMCI and naMCI (*n* = 34)

		Follow-up		Total
		MCI	AD	
Baseline	aMCI	8 (23,5 %)	11 (32,4 %)	19 (55,9 %)
	naMCI	13 (38,2 %)	2 (5,9 %)	15 (44,1 %)
Total		21 (61,8 %)	13 (38,2 %)	34 (100 %)

### Correlations whole group with objective test data

Spearman correlations were performed to compare FAI self, informant and awareness scores with objective performance measures and non-cognitive factors at both times of testing. The results including the single NTBV subtests are presented in Table 3. They show a wide variation of correlations of FAI self and informant ratings with objective performance scores (NTBV, MMSE and WST), but only 3 of them are significant. Negative correlations indicate that lower objective performance scores were in line with higher memory complaints concerning self and informant questionnaires or vice versa. Regarding self-assessment at baseline, there were two significant negative correlations with the NTBV subtests *Verbal Selective Reminding Test (VSRT) – delayed recall* and *VSRT – recognition*, which belong to the domain memory. At follow-up one significant negative correlation was found in the domain language. All significant correlation coefficients showed moderate effects (all *r*_s_: 0.30 to 0.50) according to Cohen [30].

**Table 3:** Spearman’s correlation coefficients (r_s_) between FAI self, informant and awareness scores, objective performance measures and non-cognitive factors

	FAI – BASELINE			FAI – FOLLOW-UP		
	Self	Informant	Awareness	Self	Informant	Awareness
**NTBV Domain Attention**						
AKT	0.09	0.24	−0.08	−0.34	0.07	−0.22
Digit-Symbol	0.09	0.05	0.12	0.07	−0.29	0.20
Symbols (c.I.)	0.20	−0.09	0.10	0.08	0.23	−0.28
TMT B	−0.02	−0.13	0.07	0.06	0.31	−0.24
TMT B – A	−0.07	−0.13	0.13	0.08	0.36	−0.26
**NTBV Domain Language**						
mBNT	−0.04	0.34	−0.28	−0.32	0.17	−0.16
SWT	−0.28	0.23	−0.30	−0.44*	−0.11	−0.02
**NTBV Domain Executive Function**						
TMTA	−0.14	−0.07	−0.12	0.01	0.12	−0.03
5-point	0.11	0.15	0.01	−0.16	0.09	−0.12
Stroop total/time	−0.20	0.18	−0.15	−0.12	0.09	−0.02
Labyrinth	−0.12	−0.08	0.02	−0.09	−0.23	−0.02
Interference total/time (c.I.)	0.22	0.26	−0.00	−0.28	−0.21	0.15
PWT	−0.23	0.14	−0.19	−0.16	−0.21	0.10
**NTBV Domain Memory**						
VSRT Immediate Recall	−0.08	−0.02	−0.04	0.25	−0.00	0.18
VSRT Total Recall	−0.33	−0.09	−0.08	0.02	−0.25	0.26
VSRT Delayed Recall	−0.43*	0.13	−0.29	−0.15	−0.34	0.08
VSRT Recognition	−0.48**	0.06	−0.32	−0.05	−0.15	0.27
MMSE	−0.08	−0.15	0.11	0.08	−0.34	0.50*
WST-IQ	0.09	−0.14	0.16	−0.40	0.40	−0.39
BDI	−0.05	0.00	0.04	0.10	0.03	0.07
GDS	0.05	−0.10	0.09	−0.14	0.36	−0.21
Sex	−0.11	0.06	−0.12	0.34	−0.09	0.14
Age	0.20	−0.05	0.12	−0.12	0.07	−0.23
Education	0.03	−0.22	0.08	−0.21	−0.00	−0.28

*AKT* Alters-Konzentrations-Test, *WAIS-R* Wechsler Adult Intelligence Scale – Revised, *TMTA* Trail Making Test Version A, *TMTB* Trail Making Test Version B, *PWT* Phonematische Wortflüssigkeit, *C.I.* Cerebral Insufficiency Test; *SWT* Semantische Wortflüssigkeit, *VSRT* Verbal Selective Reminding Test, *mBNT* modified Boston Naming Test, *MMSE* Mini Mental State Examination, *WST* Wortschatztest, *BDI* Beck Depression Inventory, *GDS* Geriatric depression scale

*p < 0.05. **p < 0.01

Correlation analyses of FAI awareness scores revealed a wide range of correlations with objective performance scores (NTBV, MMSE and WST). There was one significant positive correlation with the MMSE at follow up. The significant positive association indicated that overestimation was associated with lower performance on MMSE. Its correlation coefficient showed a large effect (*r*_s_ = 0.50).

Concerning non-cognitive factors, one significant positive correlation with moderate effect was found between informant scores and the Geriatric depression scale (GDS) at follow up, indicating that higher informant estimated memory complaints were associated with an increase of patient’s depressive symptoms. The demographic variables sex, age at onset and education did not reveal significant correlations regarding FAI self, informant and awareness scores.

### Total group correlations across time

Total group Pearson correlations between baseline and follow up were calculated to compare FAI self, informant and awareness scores between both times of testing. All correlation coefficients were statistically significant (all *p* < 0.01) with large effect (all *r* ≥ 0.50). See Table 4 for details.

**Table 4:** Pearson correlations between baseline and follow-up for FAI self, informant and awarenessscores; means and standard deviations

	*r*	*M*	*SD*	*n*	
Self	0.55**	2.76	0.70	32	T1
		2.81	0.59	22	T2
Informant	0.50**	3.07	0.92	34	T1
		3.03	0.98	32	T2
Awareness	0.51*	−0.33	1.14	32	T1
		−0.29	1.20	20	T2

T1 – baseline, T2 – follow-up

* *p* < 0.05. **p < 0.01

### Differences among MCI and AD patient groups across time

The two groups of MCI patients, who turned to AD and those whose remained MCI at follow-up, were examined for overall differences in FAI scores between both times of assessment by use of a Wilcoxon signed-rank test. Median scores are presented at Table 5. Wilcoxon signed-rank tests did not reveal significant differences for patients who remained diagnosed as MCI for FAI self (*Z* = −1.23, *p* = 0.22), informant (*Z* = −1.07, *p* = 0.29), and awareness (*Z* = −1.03, *p* = 0.30) ratings between both times of assessment. Those patients who turned to AD at follow-up, also did not reveal significant difference scores between baseline and follow-up for FAI self (*Z* = −0.37, *p* = 0.72), informant (*Z* = −0.73, *p* = 0.46), and awareness (*Z* = −0.73, *p* = 0.47) ratings. Regarding self and informant assessment median scores, memory complaints raised in the MCI group and decreased for those patients, who turned to AD at follow up. Concerning awareness, discrepancy scores between self and informant questionnaires decreased slightly for the MCI group and increased for those, who were diagnosed as AD at the second time. In summary, the results of the present analyses could not provide evidence that awareness changes longitudinally within the diagnosis subgroups of MCI or MCI converting to AD.

**Table 5:** Median scores of FAI self, informant and awareness ratings for MCI and AD across time

	Baseline				Follow-up			
	MCI		MCI-AD		MCI		AD	
	*Mdn*	*n*	*Mdn*	*n*	*Mdn*	*n*	*Mdn*	*n*
Self	2.75	18	3.01	4	2.81	18	2.60	4
Informant	2.88	19	3.75	13	3.00	19	3.69	13
Awareness	−0.10	16	−0.61	4	0.17	16	−1.31	4

*MCI-AD* MCI patients at baseline, converting to AD at follow-up

### Prediction of AD

Finally Receiver-Operating-Characteristics (ROC) analysis was performed to explore the relative predictive power of FAI self, informant and awareness rating to detect dementia onset in the following 2 years. The Area under the ROC curve (*AUC*) is used as an indicator of the discriminative utility of each type of awareness assessment. An area of 1 represents a perfect test, while random guessing produces an area of 0.5 [31]. FAI self, informant and awareness ratings of MCI patients at the baseline were compared with AD conversion-rates at follow-up. The results of the ROC analyses revealed an *AUC* of 0.60 for self and 0.62 for informant questionnaires indicating poor discriminative utility. Awareness discrepancy scores had similar predictive power (*AUC* = 0.42). See Table 6 for details.

**Table 6:** ROC analysis, MCI conversion to AD

	*AUC*	*S*	*p*	95 % KI	
				−	+
Self assessment	0.60	0.12	0.33	0.36	0.85
Informant assessment	0.62	0.11	0.29	0.41	0.82
Awareness	0.42	0.11	0.46	0.21	0.64

*AUC* Area under the ROC Curve

## Discussion

The main intention of the present study was to explore awareness of subjective memory assessment in patients with MCI and AD and to determine if unawareness can serve as a predictor for future conversion to AD in MCI. For this purpose, objective performance measures were compared to patient and caregiver memory reports and to awareness scores on two times of assessment with a mean interval of two years. Awareness was assessed by subtracting informant-rating from self-rating scores of the FAI questionnaire. Whole-group comparisons of awareness with objective test data revealed one significant correlation concerning overall cognitive ability (MMSE), which became significant with large effect at follow-up (*r*_s_ = 0.50). Patients with decline of cognitive performance overestimated their memory function compared to their caregivers. This outcome supports studies indicating that unawareness increases with cognitive decline [4, 11]. Whole-group comparisons between self-assessment FAI scores and the results of the NTBV revealed significant correlations with subtests of the domains memory and language. At baseline self-ratings yielded two significant associations with moderate effect to the VSRT-subtests *Delayed Recall* (*r*_s_ = −0.43) and *Recognition* (*r*_s_ = −0.48) of the domain memory, revealing intact good accordance. The VSRT assesses loss of episodic memory, which is a core diagnostic criterion for later conversion to AD [32]. Lehrner et al. [11] used the subtest *Delayed Recall* of the VSRT as a measure of objective memory in order to obtain awareness scores, because it is very sensitive to age related memory decline. Its association with self-assessment scores at baseline supports studies stating that memory complaints are supposed to be an early manifestation of memory impairment [33]. At follow-up, this correlation was not significant anymore, instead self-report correlated significantly with the *Semantic Word Fluency Test* (SWT, *r*_s_ = −0.44) of the domain language. People who had worse performance on this test complained more about loss of memory. One reason might be a decline of insight for memory impairment in patients with proceeding MCI and those who converted to AD, while insight to dysfunctions in other cognitive domains remains relatively intact in early stages of dementia [7]. Nevertheless, correlations with awareness scores, as observed in anosognosia, were not significant for these subtests of the NTBV [4]. One probable explanation is that patients, who converted to dementia at follow-up, showed mild symptoms of AD because patients scoring 23 or less on the MMSE were excluded from the study. Unlike expected, informant memory appraisals did not correlate significantly with memory tasks at any time of assessment. As mentioned above, informant follow-up ratings rather reflected decline of overall cognitive ability. Besides, difference scores at group level showed that informant FAI scores revealed better memory appraisals for the AD group at follow-up than at the baseline. Nevertheless, discrepancy scores increased, because patients overestimated their memory functions even more [7]. These findings might be influenced by several aspects like sample size, patient-informant relationship and characteristics of informants. Moreover, the current study did not include patients with moderate or severe AD, where memory deficits can easily be detected by caregivers. Neurocognitive memory tasks might identify subtle cognitive changes which might be beyond the competence of informants [10].

In the field of non-cognitive factors, sex, age and education did not reveal any effect on awareness. Whole-group comparisons of the underlying sample could not reveal evident influence of depressive symptoms on awareness. More longitudinal studies are needed to investigate effects of hyper-awareness or underestimation [8].

Another question of interest was, whether awareness changed over time in the total sample and each diagnostic group separately. Whole-group correlation analyses between baseline and follow-up showed that awareness of subjective memory assessment remained stable over time. Significant correlation coefficients with large effect (all *r*_s  _ ≥ 0.5) revealed that patients as well as their caregivers estimated patient-concerned memory functions consistently between baseline and follow-up. These results suggest that awareness of subjective memory assessment remains relatively stable in MCI and early AD patients. Separate group analyses also revealed that neither MCI nor AD-converted patients differed significantly across time regarding self, informant and awareness scores. These findings are supported by previous studies claiming that the level of awareness does not differ significantly between patients with MCI and mild AD [7]. Subjective memory complaints increased in those people who maintained the MCI diagnosis and decreased those who converted to AD. The increase of discrepancy scores within the AD group indicates a decline of awareness in AD patients who tend to overestimate their memory functions. Although not significant, these findings correspond to the significant correlates of awareness with MMSE scores. They support precedent studies indicating that decline of overall cognitive ability is associated with decrease of awareness [4, 11]. Regarding conversion rates within the MCI patient group, 11 of 13 people who turned to AD at follow-up, were diagnosed as aMCI at the baseline. Only 2 initial naMCI patients received the diagnosis of AD at the second time of assessment. These results are in line with previous studies claiming that people with aMCI are at higher risk of conversion to AD [34, 35].

One major objective of the present study was to find out the predictive power of subjective memory assessment. FAI self, informant and awareness ratings of MCI patients who converted to AD or remained MCI were subject to ROC analyses. ROC curve analyses revealed that all ratings were close to random guessing (all *AUC* < 0.65). Therefore the present study could not confirm the hypothesis that awareness of subjective memory assessment serves as predictor for future dementia. Regarding the small sample size of self and awareness scores these results have to be interpreted with caution.

The present study has some limitations. First, the sample size was generally limited due to the design of the study. Patients with moderate or severe AD were not included in the study, because they are not capable of filling in the self-assessment questionnaires. Due to the small sample size only a limited number of patients converted to AD, thus, the study was probably statistically underpowered. However, the results of the study may generate new research questions using larger clinical sample sizes. Second, as this was a clinical study, its results may not be generalizable to the general population. Third, another limitation of the study was, that depressive symptoms/psychiatric diagnosis were not assessed by psychiatric (diagnostic) interview. Finally, information about cognitive trainings, drug or other treatments against probable progression to AD between both times of testing, that might have influenced the outcome [34], was not assessed in the study.

The strengths of the study are the profound neuropsychological examination of all patients and the satisfactory sample size despite clinical limitations. Given the fact that cognitively well-preserved MCI patients usually come alone to the clinical assessment at the memory clinic, the number of participants plus informants performing pre- and post-tests is remarkable.

In conclusion, our study demonstrates that MCI patients have a relatively intact awareness of memory performance and a good self-estimation regarding episodic memory. In the longer term, cognitive decline leads to a decrease of complaints about memory loss, and to an increase of discrepancy scores between patients and their caregivers. Generally speaking, unawareness of subjective memory deficit increases to some extent in patients with incipient AD, but further long-term research is needed to clarify the relationship between awareness and AD.

## Appendix

**Table A:** Forgetfulness Assessment Inventory (FAI) self-report

Here are a few questions regarding your memory. Please answer each question by selecting the appropriate digit (1, 2, 3…). If you are not sure how to answer the question, give your best possible answer and make a remark on the left side of the page. Please do not hesitate to ask for support if you need help reading or filling in the questionnaire. How often did you have problems during the past 4 weeks remembering						
		Never	Rarely	Sometimes	Often	Very often
1	… Names of people?	1	2	3	4	5
2	… Telephone numbers?	1	2	3	4	5
3	… Faces?	1	2	3	4	5
4	… Birthdays?	1	2	3	4	5
5	… Poems?	1	2	3	4	5
6	… Book titles?	1	2	3	4	5
7	… Content of TV broadcasts?	1	2	3	4	5
8	… Shopping list?	1	2	3	4	5
9	… Directions?	1	2	3	4	5
10	… Discussion topics?	1	2	3	4	5
11	… Content of radio broadcasts?	1	2	3	4	5
12	… Content of news broadcasts?	1	2	3	4	5
13	… Arrangements?	1	2	3	4	5
14	… Prices of bread and milk?	1	2	3	4	5
15	… Numbers?	1	2	3	4	5
16	… Lyrics?	1	2	3	4	5

Adapted from [2]. Copyright 2014 by Johann Lehrner. Adapted with permission
